# Sensorimotor Learning during a Marksmanship Task in Immersive Virtual Reality

**DOI:** 10.3389/fpsyg.2018.00058

**Published:** 2018-02-06

**Authors:** Hrishikesh M. Rao, Rajan Khanna, David J. Zielinski, Yvonne Lu, Jillian M. Clements, Nicholas D. Potter, Marc A. Sommer, Regis Kopper, Lawrence G. Appelbaum

**Affiliations:** ^1^Department of Biomedical Engineering, Pratt School of Engineering, Duke University, Durham, NC, United States; ^2^Bioengineering Systems and Technologies, Massachusetts Institute of Technology (MIT): Lincoln Laboratory, Lexington, MA, United States; ^3^Department of Psychiatry and Behavioral Science, Duke University School of Medicine, Durham, NC, United States; ^4^Duke Immersive Virtual Environment, Duke University, Durham, NC, United States; ^5^Department of Electrical and Computer Engineering, Duke University, Durham, NC, United States; ^6^Athletic Department, Duke University, Durham, NC, United States; ^7^Department of Physical Therapy, Duke University, Durham, NC, United States; ^8^Olympic Team Physiotherapist, USA Shooting, United States Olympic Committee, Colorado Springs, CO, United States; ^9^Department of Neurobiology, Duke University School of Medicine, Duke University, Durham, NC, United States; ^10^Center for Cognitive Neuroscience, Duke University, Durham, NC, United States; ^11^Department of Mechanical Engineering and Materials Science, Duke University, Durham, NC, United States

**Keywords:** sensorimotor learning, full-body orienting, perception and action, immersive virtual reality, marksmanship

## Abstract

Sensorimotor learning refers to improvements that occur through practice in the performance of sensory-guided motor behaviors. Leveraging novel technical capabilities of an immersive virtual environment, we probed the component kinematic processes that mediate sensorimotor learning. Twenty naïve subjects performed a simulated marksmanship task modeled after Olympic Trap Shooting standards. We measured movement kinematics and shooting performance as participants practiced 350 trials while receiving trial-by-trial feedback about shooting success. Spatiotemporal analysis of motion tracking elucidated the ballistic and refinement phases of hand movements. We found systematic changes in movement kinematics that accompanied improvements in shot accuracy during training, though reaction and response times did not change over blocks. In particular, we observed longer, slower, and more precise ballistic movements that replaced effort spent on corrections and refinement. Collectively, these results leverage developments in immersive virtual reality technology to quantify and compare the kinematics of movement during early learning of full-body sensorimotor orienting.

## Introduction

There is a tight interplay between perception and action. The abilities to integrate information from the environment, maintain attentional focus, and swiftly formulate precise motor actions are central to daily life. Moreover, sensorimotor abilities are critical in extreme situations where success depends on the slightest of margins, such as combat, athletics, surgery, and law enforcement. Thus, there has been a concerted effort from scientists and practitioners to understand the means by which individuals learn sensorimotor skills so that this information can be utilized in applied training programs to accelerate learning (Paulus et al., 2009; Berka et al., 2010; Elliott et al., 2012; Vidal et al., 2015; Appelbaum and Erickson, 2016; Krasich et al., 2016).

The purpose of learning sensorimotor skills is to have the ability to produce, and consistently reproduce, goal-oriented movements that are specific to the task at hand (Vidal et al., 2015). Whether this involves returning an overhand serve or putting on a pair of pants, a motor plan must be implemented and adjusted based on sensory feedback. The impact of sensory information in the motor process differs between two general components of movements: ballistic and refinement (Desmurget and Grafton, 2000; Elliott et al., 2001, 2010; Urbin et al., 2011). The action trajectory is initiated in a largely ballistic manner but becomes moderated by sensory feedback at some point, especially near its end (Meyer et al., 1990; Khan and Franks, 2003). For long movements, and as per Fitts’ law, there is a balance between pre-programmed ballistic movements and feedback-mediated refinements (Fitts, 1954; Klapp, 1975; Kopper et al., 2010). Through repeated trials of reaching to static targets, when visual feedback is available, kinematics typically change so that movements are made at reduced speeds with more time spent refining movement trajectories with the available visual information (Khan et al., 2002; Heath, 2005).

While considerable progress has been made toward understanding the psychophysiological mechanisms that enable sensorimotor learning (Wolpert et al., 1995; Schmidt and Lee, 2011; Wolpert and Flanagan, 2016), most real-world actions, like catching a baseball or shooting a moving target, are extremely complex, making it difficult to model the full range of processes in native settings (Berka et al., 2010; Elliott et al., 2011). Recent advances in immersive virtual reality (VR), however, have unlocked new means by which to perform realistic sensorimotor tasks and capture granular information about the full gamut of visual, motor, and cognitive processes that underlie performance (Adamovich et al., 2009; Bideau et al., 2010; Goldberg et al., 2014; Wright, 2014).

Training in VR has been shown to translate successfully to the real-world for tasks that require learning, including procedural (Ragan et al., 2010) and motor (dos Santos Mendes et al., 2012) learning. Making the VR experience fully immersive in a “CAVE Automatic Virtual Environment” or CAVE-like system (Cruz-Neira et al., 1993), improves sensorimotor feedback as the user has full visual and proprioceptive awareness of his/her physical posture within the virtual environment. In such systems, the images are projected onto large screens that surround the user, rather than displayed through a head-mounted display. Stereoscopic glasses are used so that the virtual environment is displayed with one perspective for each eye, causing the effect of depth perception. Previous research has shown the benefits of CAVE-like systems for sports and training applications. In an early study on fly ball simulation, Zaal and Michaels (2003) were successful in replicating previous findings from the real world in a CAVE environment. Users were able to judge whether fly balls would pass behind or in front of them and could intercept fly balls (although using their foreheads rather than their hands). More recent work on a soccer goal keeper simulation concluded that anxiety markers were increased when virtual crowds were present and the virtual environment surrounded the user, underscoring the realism of the technology (Stinson and Bowman, 2014). A properly equipped CAVE-like system offers total control of many parameters of the task, as it can calculate precise timings and positions from multiple body-mounted trackers.

Precision shooting is particularly useful for studying visually guided movement because it is tightly constrained in space and time, produces feedback of performance (hits of a target) that are unambiguous and discrete, and yet requires complex psychomotor skills that demand high mental and physical coordination. Static marksmanship, in particular, has been the focus of many prior studies (Mason et al., 1990; Tremayne and Barry, 2001; Hatfield et al., 2004; Berka et al., 2008; Janelle and Hatfield, 2008; Goodman et al., 2009; Chung et al., 2011), but a number of other studies have also investigated dynamic shooting abilities (Walmsley and Williams, 1994; Mononen et al., 2003, 2007; Causer et al., 2010). By studying the interception of a moving target, such as in trap shooting, researchers can investigate refinements in the action-perception cycle of tracking the moving target as well as full-body orienting movements relative to the interception point. Participants must accurately align their gun to prepare for the launch of a clay pigeon target, then, upon release, track the moving target before pulling the trigger to intercept the pigeon that is moving away from them at speeds up to 100 km/h. The visual angle of the stimulus decreases as the target moves away from the observer, adding a cost to waiting too long to make a shot attempt. Thus, the strategy of simply moving slowly and spending more time in the refinement phase, as is done with static small targets (Kopper et al., 2010), does not suffice. Conversely, moving too quickly could incur widely erroneous movements. As such, rapid orienting is crucial, but a balance must be struck between quick and accurate movements.

Here we used the novel technical capabilities of immersive VR to establish how sensorimotor learning is manifested in movement kinematics through performance during a simulated marksmanship task. We modeled our task after Olympic Trap Shooting in consultation with a physiotherapist for the United States Shooting team (author N.D.P.) and conducted all experiments in a CAVE-like system, the Duke immersive Virtual Environment (DiVE). The methods yielded high-resolution, continuous data to supplement single-point spatial and temporal measures such as accuracy, precision, and reaction time, all collected under naturalistic conditions. We found that subjects reliably improved their performance through the course of training, resulting in a reciprocal change between the ballistic and refinement phases of each movement. Subjects elongated the duration of the ballistic phase while simultaneously improving the precision of that impulsive movement, thereby allowing refinement movements to be carried out in a more controlled fashion. Through the novel methodology proposed here, this study sets the stage for a host of future experiments that can systematically probe the kinematic and neural processes underlying sensorimotor learning.

## Materials and Methods

### Participants

Twenty individuals participated in the study (14 males). Participant ages ranged from 18 to 52 years (*M* = 24.9 years, *SD* = 8.8) with 8 individuals reporting some form of VR experience, whether it was with a CAVE-type system or HMDs. All participants self-reported that they were novices with no marksmanship experience. Most (18/20) were right-handed. Subjects voluntarily participated by reading and signing a written informed consent. No compensation was provided to subjects for participation. All experimental protocols were approved by Duke University’s Institutional Review Board [D0124/2015].

### Apparatus

**FIGURE 1:**
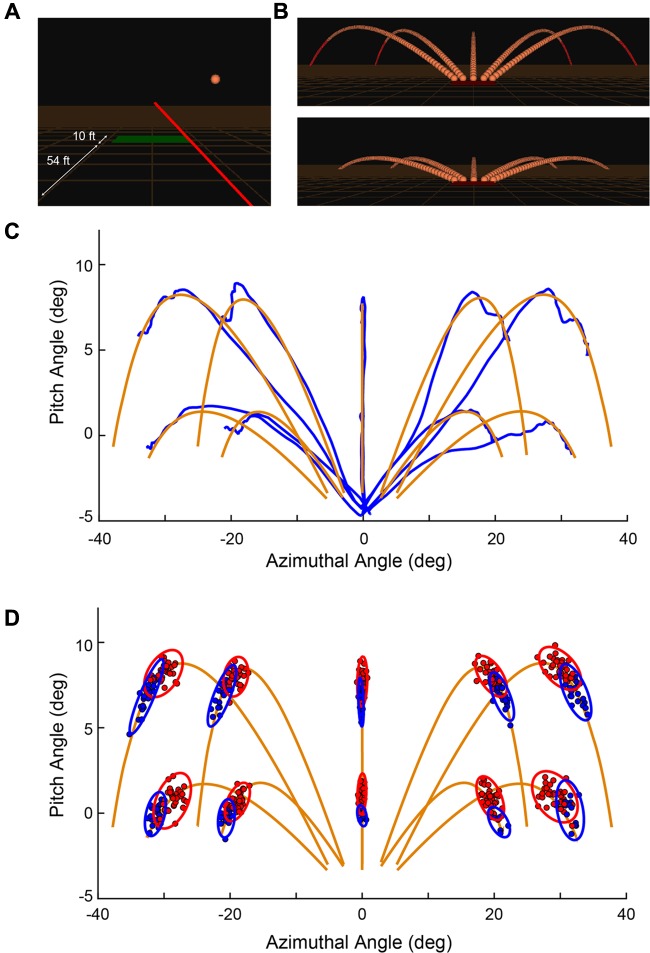
**(A)** Snapshot during a trial showing the orange spherical target in flight, the red ray emanating from the controller held in the subject’s hand (not shown here), and the green trap house from which the targets are launched. Distances are labeled for illustration and are not displayed to the subject. **(B)** All 10 possible target trajectories with all individual frames. **(C)** Representation of the target (orange) and ray motion (blue) is provided by measurements in spherical coordinates for an example set of trials in a block of data (subject 1, block 1). The azimuthal and pitch angles are computed relative to the instantaneous location of the controller. **(D)** Distribution of shots for Shot 1 (red) and Shot 2 (blue) through a session for a single subject. **(A,B)** Reprinted from Zielinski et al. (2016) with permission (© 2016 IEEE).

The experiment was conducted in the DiVE, a high-fidelity CAVE-type system on the campus of Duke University (Cruz-Neira et al., 1993). Subjects stood in the center of the 3 m × 3 m × 3 m cube, with projection on all six sides. Grid lines were added to the ground to give subjects a sense of depth and perspective. An Intersense IS-900 system (Thales Visionix, Inc., Billerica, MA, United States) was used to track head and hand movements, both of which had 6 degrees of freedom. Projectors were run at 120 Hz and provided a total resolution of 1920 × 1920 pixels per wall within the cave. Shutter glasses were used to provide active stereoscopic graphics and the effective frame rate for each eye was 60 Hz. Subjects held a controller in their dominant hand and stabilized it with their other hand mimicking a “pistol grip.” From the controller, a virtual red ray extended out into the distance (**Figure 1A**). Through the remainder of this report, we shall refer to the *Controller* as the device physically held by the subject and the *Ray* as the virtually projected pointer used to aim and intercept targets.

### Software and Data Collection

The simulation software was written in C++ and OpenGL, and utilized the VR library ‘Syzygy’ (Woo et al., 1999; Schaeffer and Goudeseune, 2003). All ray and controller movements were sampled at 60 Hz. Online, the path of the target and the movements of the ray were linearly interpolated to 20 divisions between two consecutive frames to ensure that any target interception that occurred in between samples was accurately characterized.

### Trap Shooting Task

We modeled the trap shooting task, a type of dynamic target acquisition task, on the International Sport Shooting Trap event (Official Statutes Rules and Regulations, 2013). Keeping much of the realistic feel, we adapted the paradigm for the virtual environment. In prior work, this task was tested while manipulating the frame rate and image persistence (Zielinski et al., 2016). The details of the task are explained below. In brief, a subject’s goal on a given trial was to acquire and shoot a target that was launched from behind a rectangular trap house and projected away from the subject.

To start a trial, the subject would point the ray at the trap house, which was represented as a rectangle on the ground 54 ft. (16.46 m) in front of the subject (in simulated space; **Figure 1A**). After 500 ms, the trap house changed color from red-to-green and a variable holding period (500–1000 ms) began. If the subject moved the ray from the trap house during the holding period, the delay timer was reset and did not start again until the subject moved the ray back to the trap house. Given a successful holding period, the target, represented as an orange sphere of radius 1.0 ft. (0.30 m), was launched in one of 10 possible trajectories (**Figure 1B**). These paths consisted of 5 horizontal directions relative to the center of the trap house (-45°, -30°, 0°, 30°, 45°) and 2 elevations (25.17°, 12.95°) relative to the ground plane. At a distance of 195 ft. (59.44 m) from the trap house (1.35 s), the target was considered “out of range” and could no longer be hit. In the upper elevation, in which the total flight time was 1.80 s, the target turned from orange to red if it was not hit while “in range.” In the lower elevation, the flight time was 1.35 s and if not hit, disappeared below the plane of the ground. All targets had a speed of 95.34 ft./s (29.05 m/s) and the physics of motion incorporated gravitational pull, air resistance, and lift force that were designed to mimic realistic flight times, trajectories, and distances observed in real trap events (Official Statutes Rules and Regulations, 2013).

After the target was launched, the subject aimed the ray at the moving target and pressed the trigger button on the controller to “shoot” at it. In **Figure 1C**, one subject’s single trial aiming movements (blue) are shown in comparison to each of the 10 possible target trajectories (orange). Targets were launched from the top edge of the trap house, which was 5° below the horizontal plane of the ray. On each trial, the subject was allowed up to 2 attempts to hit the target. **Figure 1D** shows an example distribution of Shot 1 (filled red) and Shot 2 (filled blue) from a single subject. Principal component analysis was used to construct ellipses around the data indicating 95% confidence limits (Messier and Kalaska, 1999). If the ray was contacting the target at the time of a shot, the target would visually shatter, a shattering sound would be played, and the trial would immediately terminate and be counted as a success. If the target was not successfully hit within the allotted time, the target continued its trajectory and disappeared upon passing beneath the plane of the ground. On each trial, a trigger press elicited a short “click” sound for Shot 1 and (if taken) Shot 2, but no sound after that. At the end of each trial, and when participants were ready to initiate the next trial, they aimed the ray back over the trap house and the shot sequence described above was repeated.

Performance data was analyzed for Shot 1 through measurement of two conventional variables, *Accuracy* (hits or misses) and *Response Time* (elapsed time from target launch to Shot 1). Note, the latter differs from Reaction Time (discussed below), which was based on the orienting movement rather than the trigger press. *Trial Success* was the overall hit rate of completed trials regardless of number of shots taken. We included this variable to maintain ecological validity of the technique, since the International Sport Shooting Trap event allows two shots at each target. In our study, Shot 2s were infrequent and heterogeneous across target trajectories, so although they contributed to Trial Success, they were not analyzed in detail.

The target launch directions were designed to be symmetric about the midline to ensure similarity in experience between right- and left-handed participants. To standardize the analysis, a directional convention is adopted that preserves the movement of the target relative to the participants’ handedness. The five target directions are referred to as Far Contralateral, Contralateral, Center, Ipsilateral, and Far Ipsilateral, with reference to the hand holding the controller. Thus, for the 18 right-handed subjects, Contralateral is leftward, while for the 2 left-handed subjects, it is rightward. There are no characteristic differences in movement behaviors between the left- and right-handed subjects and no subject had shot accuracy performance beyond 2 standard deviations of the mean, therefore, in subsequent population analyses, the data is collapsed into Contralateral and Ipsilateral conditions across all 20 subjects.

Each subject performed 7 blocks of 50 trials each. All 10 target trajectories were presented 5 times within each block in a randomized manner. On average, subjects took 187.20 s (*SD* = 15) to complete each block. To allow for an adequate period of rest between blocks, all subjects performed the experiment in pairs. While one participant was performing the task, the other was taking a break. The pairs alternated performing the blocks through the session thereby mitigating fatigue. The subject that was not actively performing the task sat outside of the DiVE and was not able to observe the other participant perform the task.

### Movement Analysis

**FIGURE 2:**
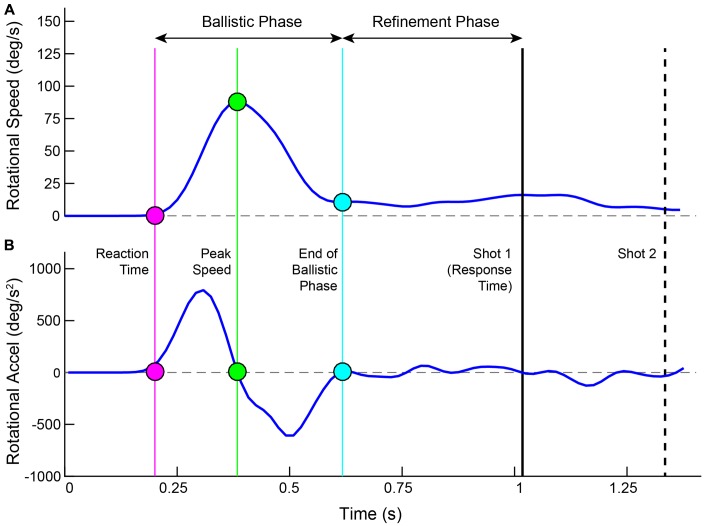
Plotted are the ray rotational speed **(A)** and acceleration **(B)** for an example trial. Time is aligned to the launch of the target. Filled circles show the Reaction Time (magenta), Peak Speed (green) and End of Ballistic Phase (cyan). These three time points are drawn in the acceleration trace as well. The shot 1 Response Time is shown as a solid black line and the Shot 2 time is shown as a dashed black line.

Movements were calculated in 3 separate coordinate frames. The direction that the ray was pointed in and the rotation of the head were each computed in independent spherical coordinates. The movement of the controller was measured in Cartesian space. Rotational speed was computed as the angular displacement of the ray between successive frames. Through this report, we refer to it as *Ray Rotation (RR).* Raw speeds were passed through a third order low-pass FIR filter, cutting off at 0.2 of the normalized frequency. Acceleration was computed on the filtered speed trace and received no additional filtering. Offline, both the speed and acceleration were linearly interpolated from 60 to 1000 Hz. **Figure 2** illustrates the rotational speed and acceleration for an example trial along with several time demarcations calculated from the movement and shot actions. For each trial, the *Peak Speed* was calculated as the highest point in the trace, which also corresponds to the zero crossing in the acceleration trace. The *Reaction Time* was the time from target launch to the start of movement acceleration, found by stepping earlier in time from Peak Speed to the time point corresponding to 5% of that speed (van Donkelaar and Franks, 1991; Chua and Elliott, 1993). Stepping forward in time from Peak Speed, the end of movement deceleration was found similarly (5% of the peak).

The entire movement was separated into two phases – a *ballistic phase* and a *refinement phase* (Elliott et al., 2001). The ballistic phase was the time range from start of acceleration to end of deceleration (Meyer et al., 1988; Abrams et al., 1990). The refinement phase started at the end of the ballistic phase and concluded when the first shot was taken (Beggs and Howarth, 1972; Crossman and Goodeve, 1983; Meyer et al., 1988, 1990). The ballistic phase was further dissected into two segments – its rising phase (from start of acceleration to Peak Speed) and its falling phase (from Peak Speed to end of deceleration).

In addition to the rotation of the ray, there was translational movement of the controller that the subjects held in their hand. This Cartesian motion is referred to as *Controller Translation (CT)* through this report. The same metrics of movement that were computed in angular coordinates were computed in linear coordinates. The onsets of movement, times of peak speed, and end of ballistic phases occurred at roughly the same times. Though highly correlated, there was some variability across each trial.

Angular error was defined as the angle between the directional point of the ray and the direction between the controller and the target. We include in our analyses the instantaneous angular error at the end of the ballistic phase.

The movement of the subject’s head was also tracked and the rotational speed of head movements was computed on a trial-by-trial basis. *Head Rotation (HR)* was calculated in the same manner as *Ray Rotation*, but was not split in ballistic and refinement phases. Translational head movements were negligible and excluded from analyses.

### Statistics

Trials were excluded if subjects did not take a single shot or took the first shot when the target was out of range (120 trials, 1.86%). Trials were also excluded if subjects initiated movements too quickly, less than 16.667 ms (13 trials, 0.19%), or completely failed to move the ray from the trap house (7 trials, 0.01%).

We ran a three-way Repeated Measures Analysis of Variance (ANOVA) with the following factors: 2 target elevations (*Elevation*), 5 target directions (*Direction*) and 7 blocks (*Block*). The data were tested for sphericity and whenever the assumption was violated, the Greenhouse–Geiser correction on the degrees of freedom was used. Statistical analyses were meant to test the hypotheses that there were within-subject changes through learning. In the figures presented in the Section “Results,” traces are averaged across subjects for visualization purposes only. Error bars are excluded to avoid depiction of inter-subject distributions which are a result of individual variability, and are not essential to the within-subject statistical tests in this study.

There were many trials in which a second shot was not taken. This could happen in cases where the first shot was successful rendering the second shot unnecessary, if the second shot was only taken after the target was out of range, or if the second shot was not taken at all. Because there were a high percentage of trials, 62.6%, in which Shot 2 was not taken, and these were not uniform across the 10 target trajectories, we focused our analyses on Shot 1 and included Shot 2 performance only into the metric Trial Success.

## Results

Behavioral data collected during this simulated marksmanship task comprised both traditional measures of Shot Accuracy and Response Times, as well as novel high-precision information about the movement kinematics that transpired over the course of a trial. In the following sections, we present first the results describing Shot Accuracy, followed by results describing the changes in movement kinematics observed through Blocks.

### Shot Accuracy

**FIGURE 3:**
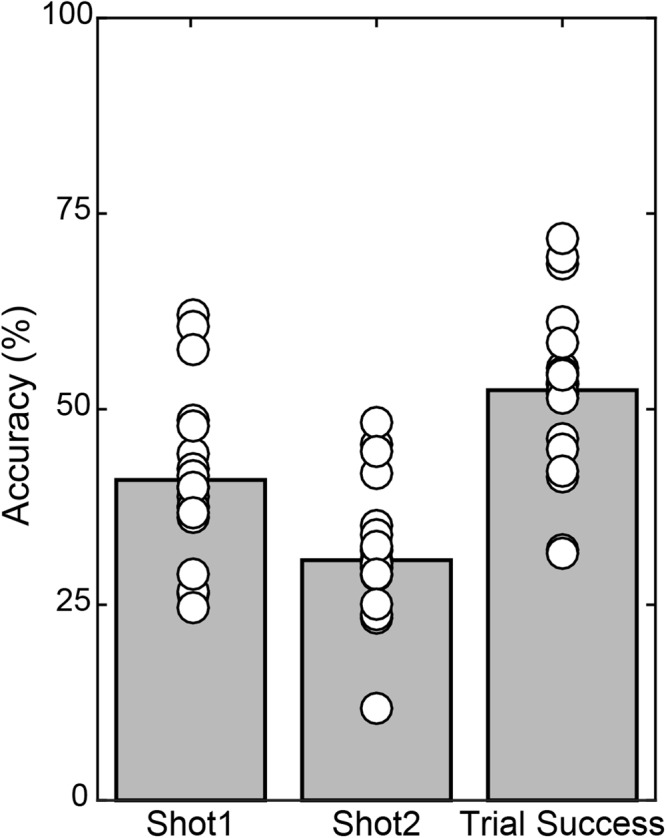
Mean accuracy (gray bars) and individual subject accuracy (white circles) are shown for Shot 1 and Shot 2 relative to number of those shots taken, and overall Trial Success, relative to all trials included in the analyses.

Across the 20 subjects, each individual performed 7 blocks of 50 trials leading to 7000 total trials in the study, minus 140 trials excluded as described in Methods. In the remaining 6860 trials, all included a Shot 1 and 2568 included a Shot 2, with 41.0% accuracy on Shot 1 (**Figure 3**). In the 59.1% of overall trials in which Shot 1 missed, the subject followed with a Shot 2 63.4% of the time, which had 30.7% accuracy. Thus Shot 2 yielded a hit on 11.49% of all trials. Summing Shot 1 and Shot 2 accuracy yielded an overall Trial Success of 52.4%.

**FIGURE 4:**
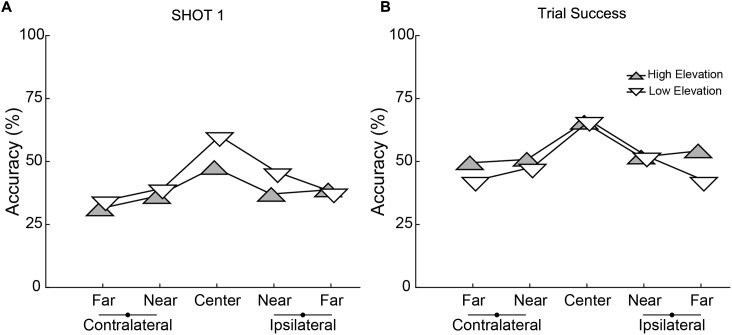
Accuracy for the two elevations and five directions for **(A)** Shot 1 and **(B)** Trial Success. High elevations are represented with gray, upward pointing triangles and lower elevation with white, downward pointing triangles. Each point represents the mean performance across 20 subjects. Lateralization is relative to each subject’s dominant hand (which held the controller).

Performance depended on the parameters of target movement. Separate 2 × 5 × 7 (Elevation × Direction × Block) ANOVAs performed on the accuracy of Shot 1 Accuracy and Trial Success revealed main effects of Elevation for both (Shot 1 Accuracy: [*F*(1,19) = 9.288, *p* = 0.007], Trial Success: [*F*(1,19) = 7.760, *p* = 0.012]). Shot 1 was less accurate for the higher elevation (**Figure 4A**) but Shot 2 made up for this as indicated by better overall Trial Success for the higher elevation (**Figure 4B**). There was also a significant main effect of Direction for both accuracy measures (Shot 1 Accuracy: [*F*(4,76) = 21.010, *p* < 0.001], Trial Success: [*F*(4,76) = 25.684, *p* < 0.001]) with the best performance in the central trajectory and nearly symmetric decreases in accuracy for the flanking directions. For both these metrics of shooting accuracy, there was a significant interaction between Elevation and Direction (Shot 1: [*F*(4,76) = 3.394, *p* = 0.013], Trial Success: [*F*(4,76) = 3.384, *p* = 0.013]). When considering Shot 1, *post hoc* comparisons showed that accuracy was significantly better in the low elevation for the central (*p* < 0.001) and near ipsilateral (*p* = 0.018) directions, and was not different between elevations in the other three directions. For the numerical values of the group data shown in **Figures 4A,B**, see Supplementary Table 1.

**FIGURE 5:**
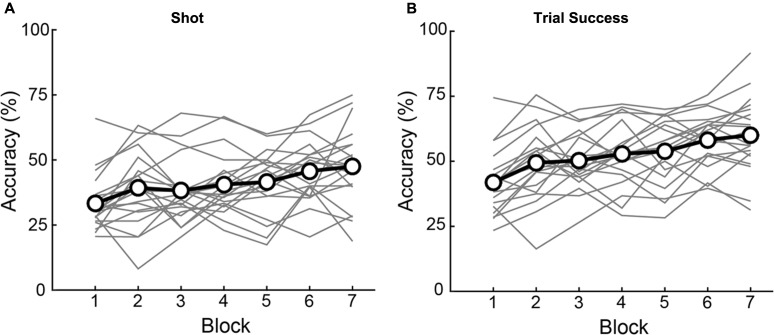
Individual subject (gray lines) and group average (black lines with open circles) accuracy over blocks. Significant improvements were observed for both Shot 1 accuracy **(A)** and Trial Success **(B)** over blocks. Of the 20 individual subjects, seventeen had higher Shot 1 accuracy in the last block as compared to the first, while 18 had higher Trial Success performance on the last block as compared to the first.

Performance depended, as well, on training, which we quantified as changes in accuracy across successive blocks of trials. As illustrated in **Figure 5**, there was a main effect of Block on both Shot 1 Accuracy [*F*(4.081,114) = 7.617, *p* < 0.001] and Trial Success [*F*(6,114) = 16.167, *p* < 0.001]. Across the 7 blocks, subjects showed a significant linear improvement (within-subjects contrasts for Accuracy and Trials Success, *p* < 0.001; no significant 2nd or 3rd order polynomial terms). There was no difference between the number of Shots 1 and 2 per block (Shot 1 number: [*F*(6,133) = 1.25, *p* = 0.286], Shot 2 number: [*F*(6,133) = 0.56, *p* = 0.765], implying that increased performance is not an artifact of taking more shots. There was no interaction of Elevation or Direction with Block on Shot 1 Accuracy (Elevation: [*F*(6,114) = 0.395, *p* = 0.881], Direction: [*F*(24,456) = 1.329, *p* = 0.138]) or Trial Success (Elevation: [*F*(6,114) = 0.304, *p* = 0.934], Direction: [*F*(6,114) = 0.304, *p* = 0.138]) indicating that learning was uniform across trajectories. The end result was an improvement of 14.1% in Shot 1 Accuracy and 18.2% in Trial Success during training (calculated as group data in last block minus first block). For the numerical values of the group data shown in **Figure 5**, see Supplementary Table 2.

In sum, accuracy varied significantly but mildly as a function of trial geometries (the various trajectories we used to minimize prediction of flight paths), but the critical result was that according to both measures, Shot 1 Accuracy and Trial Success, subjects experienced significant, steady increases in task performance due to training across successive blocks. Hence, the task elicited sensorimotor learning. Next, we go past these traditional measures of performance to analyze changes in movement kinematics that accompanied this learning.

### Movement Kinematics

**FIGURE 6:**
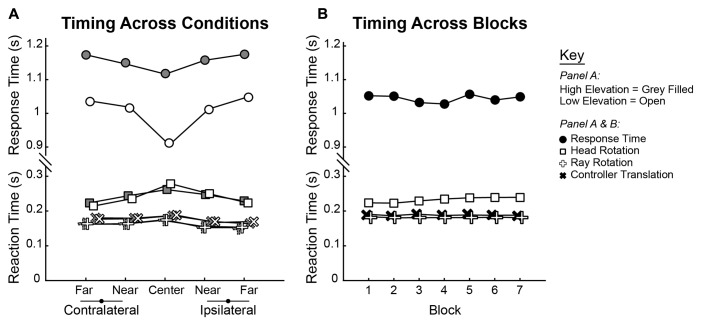
Group average Reaction and Response Times plotted across **(A)** Elevations and Directions and **(B)** across Blocks. Reaction Times are split by those computed by rotations of the head (square) and ray (plus sign) as well as translation of the controller (“x” sign). For each measure, statistics were computed in ANOVA as within-subjects trends, and group means are shown for each condition.

To understand changes in movement kinematics that occurred over blocks, three types of movements were analyzed. The first two correspond to movements of the hand that are split into Ray Rotation (RR) and Controller Translation (CT). The third type of movement was Head Rotation (HR). For each of these calculations, two time intervals were calculated relative to target launch time. Reaction Time was the latency to the start of movement, while Response Time was the latency to Shot 1. The average group Reaction and Response Times are shown across conditions in **Figure 6A**, while group averages across blocks are shown in **Figure 6B**. As was done for shot and trial accuracy, separate 2 × 5 × 7 (Elevation × Direction × Block) ANOVAs performed on parameters of the movement kinematics. This was done for RR, CT, and HR Reaction Times and Response Times.

Qualitatively, both Reaction and Response time varied across trajectories (Elevation and Direction). Reaction Times were slowest for the Center trajectories as compared to the Near and Far ones, but the inverse was true for the Response Time which were fastest for the Center trajectories (**Figure 6A**). Comparing elevations, Response Times were markedly faster for the lower elevations. However, Reaction and Response Times were essentially flat through training (**Figure 6B**). Quantitatively, the results of all tests are as follows. For all three measures of Reaction Time, there were significant main effects of Direction (RR: [*F*(4,76) = 17.639, *p* < 0.001]; CT: [*F*(4,76) = 8.818, *p* < 0.001]; HR: [*F*(4,76) = 5.132, *p* < 0.001]). In each case, the initiation of movement was slowest in the central direction and faster for the more eccentric trajectories. There was no main effect of Elevation on any of the Reaction Time measures (RR: [*F*(1,19) = 0.049, *p* = 0.827]; CT: [*F*(1,19) = 0.211, *p* = 0.651]; HR: [*F*(1,19) = 0.070, *p* = 0.795]). Across Block, there was no main effect of either of the hand-based Reaction Time measures (RR: [*F*(6,114) = 0.701, *p* = 0.649]; CT: [*F*(6,114) = 0.968, *p* = 0.450]) but there was a significant increase in the head-based Reaction Time measure [*F*(6,114) = 4.448, *p* < 0.001]. For the Response Time, ANOVAs revealed significant main effects of Direction [*F*(2.539,76) = 21.010, *p* < 0.001] and Elevation [*F*(1,19) = 133.656, *p* < 0.001] but not of Block [*F*(6,114) = 2.001, *p* = 0.071].

**FIGURE 7:**
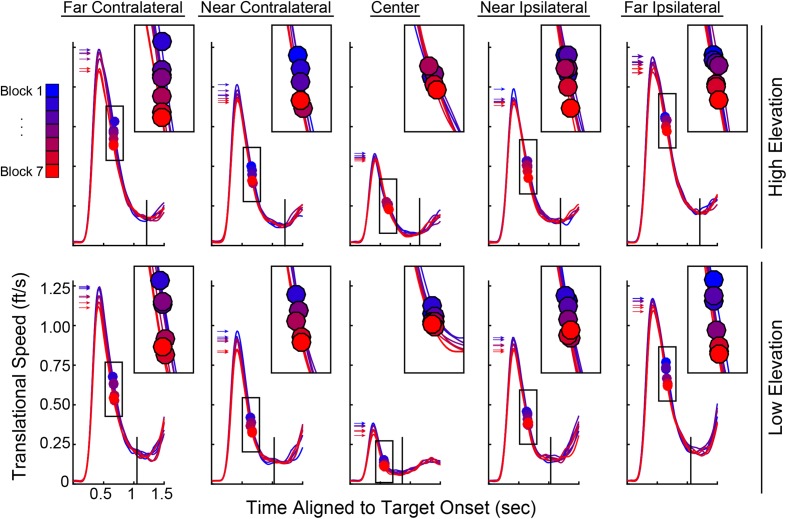
Ray rotational speed traces pooled across subjects and averaged for each of the 10 target trajectories. Top row, High Elevation; bottom row, Low Elevation; leftmost, Far Contralateral and rightmost, Far Ipsilateral. Within each panel, there are seven speed profiles with each representing one block of 50 trials. Gradient from blue to red corresponds to Block 1 to Block 7 to illustrate training effects. Insets at upper right of each panel show magnification around the filled circles (same boxes as those on the respective speed profiles), which denote the end of the ballistic phase (cf. cyan dots in **Figure 2**). Arrows in each panel point to the Peak Speed on each speed profile (cf. green dots in **Figure 2**) and black vertical lines show the average Response Time.

**FIGURE 8:**
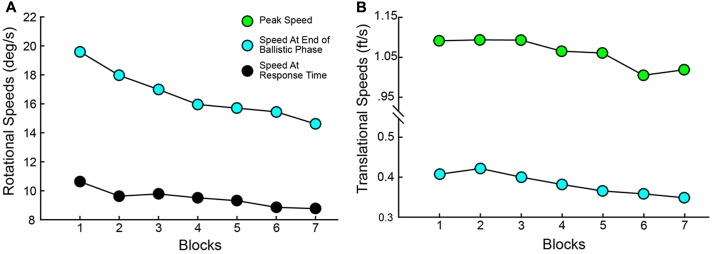
Speed parameters change significantly through training. **(A)** Speed at End of Ballistic Phase and at Response Time vs. Blocks for Ray Rotation. **(B)** Speed at End of Ballistic Phase and Peak Speed for Controller Translation. These are averages across subjects. Color codes match those in **Figure 2**.

Variables describing the movement patterns also differed across target trajectories and training. Peak RR velocities were significantly different by Elevation [*F*(1,19) = 14.811, *p* < 0.001] and Direction [*F*(1.587,76) = 259.593, *p* < 0.001] with higher velocities in the upper Elevation and more eccentric Directions (compare overall speed profiles across the panels in **Figure 7**). In addition, the more eccentric trajectories had higher speeds at the time the Ballistic Phase ended (compare the boxes on the speed profiles across the panels in **Figure 7**). There was a main effect of Block on the time at which the Ballistic Phase ended (see filled circles, magnified in the upper right inset of each panel, in **Figure 7**) when computed on the RR speed [*F*(3.509,114) = 8.126, *p* < 0.001]. The duration of the Rising Phase of movement speed during the ballistic period did not change with Blocks [*F*(1.824,114) = 0.943, *p* = 0.392] and neither did the Peak Speed [*F*(2.407,114) = 0.363, *p* = 0.736]. However, the duration of the Falling Phase in RR speed shortened through Blocks [*F*(3.100,114) = 5.863, *p* = 0.001]. With these variables, there were no interactions between Elevation or Direction with Block. Similar movement patterns were also observed in CT measurements across blocks, with an additional reduction in CT Peak Speeds (see Supplementary Figure 1). The RR speed at the End of the Ballistic Phase [*F*(3.444,114) = 8.126, *p* < 0.001] and the RR speed at the Response Time [*F*(2.203,114) = 9.227, *p* < 0.001] reduced through blocks (**Figure 8A**). Further, the CT Peak Speed [*F*(2.630,114) = 5.608, *p* = 0.003] and the CT speed at the End of the Ballistic Phase [*F*(2.429,114) = 3.216, *p* = 0.006] decreased through blocks (**Figure 8B**).

**FIGURE 9:**
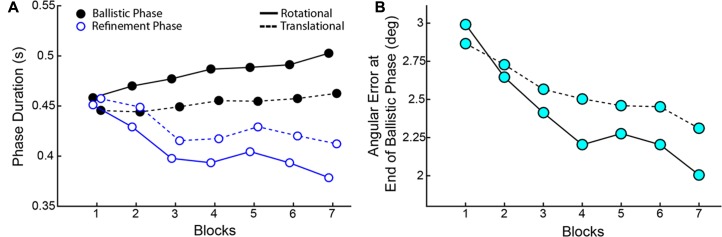
**(A)** Tradeoff between the Duration of Ballistic Phase (black) and the Duration of Refinement Phase (blue) over blocks. **(B)** Angular errors at the End of the Ballistic Phase. In both panels, solid lines correspond to measurements made on RR speeds and dashed lines correspond to CT speed measurements. Data show averages across all subjects.

As training progressed, though Reaction Times and Response Times did not change across blocks, there was a striking tradeoff between the Duration of the Ballistic Phase and the Duration of the Refinement Phase (**Figure 9**). ANOVAs showed a main effect of Block on both duration metrics for RR and CT. Duration of the Ballistic phase increased through Blocks (RR: [*F*(6,114) = 5.836, *p* < 0.001], CT: [*F*(6,114) = 2.860, *p* = 0.014) and conversely, the Duration of the Refinement Phase decreased (RR: [*F*(6,114) = 7.374, *p* < 0.001], CT: [*F*(6,114) = 3.216, *p* = 0.006]). The ballistic duration as measured from RR speeds were longer than those measured from CT ones owing to later Reaction Times (Wilcoxon Signed-Rank, *p* < 0.001), but this is compensated for by shorter durations of the refinement phase (Wilcoxon Signed-Rank, *p* < 0.001). Not only were the ballistic phases elongated in time, the movements became more accurate. The Angular Errors at the end of the ballistic phases significantly reduced through Blocks (RR: [*F*(6,114) = 9.703, *p* < .001], CT: [*F*(6,114) = 6.365, *p* < 0.001]).

**FIGURE 10:**
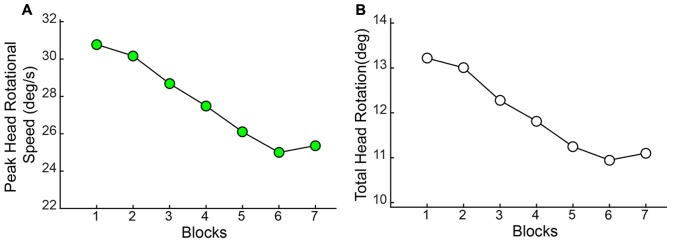
Angular rotation of the head (HR) reduces with practice. Both **(A)** Peak Speed and **(B)** Total Angular rotation decreased significantly through blocks.

As observed with the changes in hand/arm movement kinematics, similar changes were observed in the rotational kinematics of head movements through the course of the session. Both the Peak Speed of HR [*F*(2.709,114) = 12.013, *p* < 0.001] and the Total Angular Rotation of the head [*F*(2.877,114) = 11.496, *p* < 0.001] significantly decreased through blocks (**Figure 10**).

## Discussion

We tested sensorimotor performance during a simulated trap shooting task deployed in a fully immersive virtual environment. As targets were launched, subjects used a hand-held controller to track and acquire (“shoot”) them. We measured hand and head movements, along with end-point performance, to characterize motor kinematics during the entire motion of a trial. Through the course of 350 trials of training, which lasted roughly an hour, subjects improved their accuracy by an average of 18.2%. Hand movement Reaction Times and shot Response Times did not change, but parameters of the movement kinematics did. The most prominent change was that the ballistic phase of movement became longer and slower, and ended closer to the actual target. The refinement phases consequently became shorter and shots were taken with the controller moving at lower speeds. As such, this experimental approach provides rich information about motor learning in an environment that mimics an ecologically valid setting while preserving the high-fidelity control needed for experimentation.

### Sensorimotor Learning as Reflected by Changes in Movement Kinematics

Psychometric studies in which learning is measured through quantitative movement analysis have revealed regularities at the behavioral level suggesting organizational principles for learning (Corcos et al., 1993; Jaric et al., 1993; Pruszynski and Scott, 2012; Diamond et al., 2015; see Wolpert and Flanagan, 2016). Reinforcement learning models revolve around the stringing of motor routines that allow humans to quickly adapt their movements to variations of the situation without the need to re-learn complete movements (Schmidt and Wrisberg, 2000). As Vidal points out, “humans are able to detect their errors very quickly and (in most of the cases) are fast enough to correct them before they result in an overt failure” (Vidal et al., 2015). This has important implications for where feedback can alter the ballistic/refinement kinematics. In turn, the current findings indicate that through the course of practice, novice participants show dramatic modifications in their movement kinematics. The observation that these changes are most strongly reflected in the balance of ballistic versus refinement movements, points to this inflection as a key stage wherein visual feedback can alter performance and guide learning (Khan and Franks, 2003). With visual information continuously available through the trial, one would expect that larger portion of the movement would be spent in the feedback-based refinement phase (Khan et al., 2002; Heath, 2005). In this 3D simulation, however, the targets become smaller the further they travel, making intercepting them harder at longer delays. Thus, subjects elongate the ballistic phase of movement to reduce the cost of making an attempt later into the trial. Further, we observe that the Peak Speed of movement and the Duration of Falling Phase decreased through training for all target trajectories. These findings indicate that with practice, subjects execute more stable movements with less acceleration and lower speeds through the refinement phases.

During dynamic tasks like marksmanship, arm, head and eye movements need to be coordinated as visual information is transformed into motor output (Hayhoe et al., 2003). Movement of the eyes and head create additional spatial and temporal uncertainty that would lead to errors in movement. Though eye movements were not recorded in this study, our results show that, through training, the Peak Speed of head rotation and the Total Angular Rotation decreased across blocks. These findings again suggest slower and more refined movements and are consistent with prior work showing the importance of steady vision during marksmanship and other coordinated interception tasks (Land and McLeod, 2000; Causer et al., 2010; Mann et al., 2011).

A key result in our study is the higher accuracy overserved in the central trajectory and more so for the lower elevation. Subjects performed more accurately when they were required to move less in these center trajectory conditions. Acquiring a target requires precision in three categories: horizontal movement, vertical movement, and shot timing. For the central trajectory, the problem is simplified in that there is little horizontal movement and for the lower trajectory, there is additionally less vertical movement. The combination of these conditions yields less body movement (i.e., lower speeds) and less head movement (i.e., steady vision). These results have practical implications for the international shooting sport federation because they provide a method of systematically varying the levels of difficulty either during training exercise or during competitions. The heuristic being that the greater the amount of elicited movement of the body, the more difficult the task of marksmanship. With further study in simulated environments, this relation between extent of target movement and task difficult can be modeled and so used to quantify or categorize the expertise of a marksman.

### Simulation and Sensorimotor Training

Simulation-based training, such as the protocol undertaken in this research, has gained increasing use in applied settings. This is particularly true in applications where it is valuable to simulate stress so that individuals can overcome anxiety that may result in decreased performance within the safe context of a simulated activity. Over the past several years, academic research has moved toward the validation of these training techniques for the learning of transferable skills. This research has provided empirical support for the value of simulation training in domains such as surgery (Sturm et al., 2008) and manufacturing (Mujber et al., 2004). Moreover, there has been an increasing use of sports-specific virtual reality simulations for sports such as tennis (Xu et al., 2009), ping pong (Knoerlein et al., 2007), billiards (Gourishankar et al., 2007), archery (Göbel et al., 2010), handball (Bolte et al., 2010), baseball (Gray, 2002; Fink et al., 2009; Zaal and Bootsma, 2011), and rugby (Miles et al., 2012).

Beyond the use of simulation-based training, there has been an increased interest in visual-based training interventions that target sensorimotor processing to improve subsequent performance. Such approaches are based on demonstrations that certain elements of visual perception [e.g., enhanced gaze tracking for hitting a baseball (Mann et al., 2013)] and attention [e.g., attentional profiles matching the distribution of sporting activities; (Hüttermann et al., 2014)] are enhanced in experts. Moreover, this knowledge has led to a host of new sports vision training approaches that target these critical abilities (reviewed in Appelbaum and Erickson, 2016). For example, empirical research has shown benefits of stroboscopic visual training (Appelbaum et al., 2011, 2012; Wilkins and Gray, 2015), perceptual learning (Deveau et al., 2014a,b), and eye tracking interventions (Oudejans et al., 2005; Moore et al., 2012) toward enhancing athletic and competitive performance. In a previous research project from our group, these elements of simulated marksmanship training and visual training were combined, demonstrating preliminary evidence toward the integration of these approaches (Zielinski et al., 2016).

### Limitations and Future Directions

The current study investigated learning in novice participants tested on a novel shooting task. Despite the short duration of training during the experimental session, we saw marked improvements in performance across the population of participants. While typical investigations of skill acquisition show linear increase in performance initially followed by a plateauing of the learning rate in time (Newell and Rosenbloom, 1981; Heathcote et al., 2000), the present study shows predominantly linear improvements through Blocks (see **Figure 5** and associated text). Additionally, no significant interactions of Elevation and Direction were seen with Block, indicating that though performance differed across the horizontal trajectories and vertical elevations, learning was uniform across these conditions. These results could be attributed to the short duration of the experimental session (∼1 h). Further investigation is needed to assess changes in sensorimotor learning as novices train for longer periods of time and across multiple sessions.

Because this program of research is aimed at developing ecologically valid measures of sensorimotor learning, it will be important to test and validate these findings in expert marksmen. By comparing how performance in this simulation corresponds to actual trap shooting abilities in experienced individuals, we can ascertain how the simulation can be improved to more accurately reflect external environments and also determine how training within the simulation can translate to real world improvements. Motor strategies can be contrasted between novices and expert marksmen to determine how these groups differ in simulated shooting performance. Toward the goal of analyzing expert movement, we will integrate a data acquisition system that will sample head and hand movement data above 60 Hz so that nuanced differences in movement can be discerned despite the high movement speeds. Further, novel trajectories can be randomly interleaved during the experiment to further probe generalized target acquisition strategies learned by both novices and experts (Pearson et al., 2010; Krakauer and Mazzoni, 2011).

Though the paradigm used in this study mimics many features of real trap shooting, there are still gaps between the simulated task and real-world shooting. In order to improve the ecological validity of this simulation, future versions could provide more natural background scenes, utilize a more realistic shotgun device form factor, remove the ray from the view of the subjects, add the physics of realistic spreading of shotgun pellets, and insert the brief delay that occurs as pellets travel from gun to target. Given the differences within the virtual environment, any participant, novice or expert, would have to learn to perform the mechanics of the task in VR, in addition to the sensorimotor learning undergone through training. These two facets of learning are currently intertwined. Future work could therefore address the link between VR and reality, which would help refine the type of learning observed during training as well as aid in evaluating the efficacies of training within VR and the translation to a natural setting.

On roughly 60% of the trials, subjects did not take a second shot (Shot 2). Thus, Repeated Measures ANOVAs were not performed on Shot 2. Rather, Shot 2 was inherently captured within the measure of Trial Success. There were additional difficulties in interpreting the success of Shot 2. For example, metrics such as Reaction Time, Peak Speed, Duration of Ballistic and Refinement phases, and others, were shown to have significant influences on the outcome of Shot 1, but their tie to the outcome of Shot 2 is indirect. To best assess the factors influencing the outcome of Shot 2, additional analyses are needed that focus on the kinematic variables measured after Shot 1 occurs. Future work would include extending the duration of the target flight, beyond what is typically observed in real competitions, which would allow subjects more opportunity to take a second shot. A greater number of trials would also allow for a more in-depth analysis into the components of corrective behavior.

## Conclusion

Sensory-guided motor orienting actions are one of the most common and important operations that humans perform. Since the turn of the 20th century when Woodworth (1899) began testing the psychometric properties of voluntary movements, there has been considerable interest in understanding the mechanisms by which individuals perform and gain proficiency at skilled movements. In recent years these efforts have been greatly aided by new technology, and in particular, immersive VR which allows for both the precise tracking of movement kinematics and the rigorous implementation of naturalistic tasks that capture the challenges of real-world activities. As demonstrated here, training in our full-body orienting task – a simulation of trap shooting – was accompanied by gradual, robust enhancement of ballistic action and concurrent diminishment of refinements that are likely feedback-moderated. These dynamic changes occurred within a stable temporal window between nearly constant Reaction and Response Times. One concern about using more naturalistic tasks, as done here, is that behaviors will be so complex and variable as to be uninterpretable. The systematic, time-constrained yet highly dynamic training effects we found here, however, suggest great potential for such tasks to shed light on naturalistic behaviors and, ultimately, their neural basis through the addition of physiological monitoring (e.g., EEG, fNIRS, TMS) during immersive VR experiments.

## Ethics Statement

All subjects gave written informed consent in accordance with the Declaration of Helsinki. The protocol was approved by the Duke University Institutional Review Board [D0124/2015].

## Author Contributions

HR, DZ, NP, MS, ReK, and LA conceived the work and wrote the manuscript. HR, RaK, DZ, YL, and LA carried out the experiments. HR and RaK performed the data analysis. JC provided feedback on analysis and the manuscript.

## Conflict of Interest Statement

The authors declare that the research was conducted in the absence of any commercial or financial relationships that could be construed as a potential conflict of interest.
