# Supplementary material for: Sensorimotor Learning during a Marksmanship Task in Immersive Virtual Reality
Source: Front Psychol. 2018 Feb 6;9:58. doi: 10.3389/fpsyg.2018.00058 (PMC5808129; doi:10.3389/fpsyg.2018.00058)
Supplement: Supplementary file 1 [file Image_1.pdf]

# *Supplementary Material*

## Sensorimotor learning during a marksmanship task in immersive virtual reality

Hrishikesh M. Rao, Rajan Khanna, David J. Zielinski, Yvonne Lu, Jillian M. Clements, Nicholas D. Potter, Marc A. Sommer, Regis Kopper, L. Gregory Appelbaum

## 1 Supplementary Figures

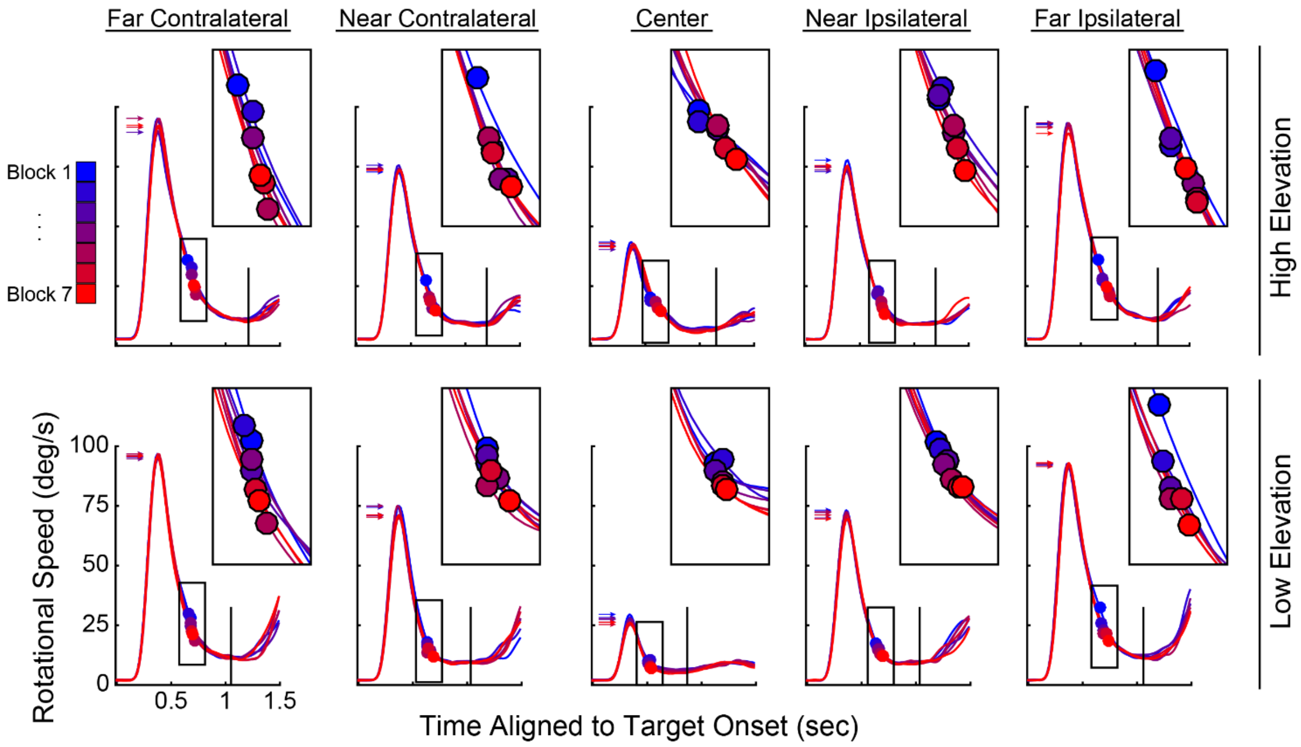


**Supplementary Figure 1.** Controller Translational speed traces pooled across subjects and averaged. Layout follows the conventions of Figure 7. ANOVAs performed across Elevation and Direction revealed main effects of Peak Speed (Elevation: [$F(1,19) = 52.435$, $p < .001$]; Direction: [$F(1.587,76) = 211.322$, $p < .001$]), matching the results observed from RR speeds albeit with lower speeds in the center trajectory and in the lower elevation. Over blocks, Peak Speeds decreased significantly [$F(2.603,114) = 5.608$, $p < .001$], as did the time at which the Ballistic Phase ends [$F(6,114) = 3.216$, $p < .001$].

## 2 Supplementary Tables

| | Shot 1 Accuracy (%) | | Trial Success (%) | |
|---|---|---|---|---|
| **Target Directions** | High Elevation | Low Elevation | High Elevation | Low Elevation |
| Far Contralateral | 31.31 | 34.52 | 49.49 | 42.44 |
| Near Contralateral | 36.24 | 39.36 | 50.81 | 47.78 |
| Center | 47.67 | 60.09 | 65.57 | 66.22 |
| Near Ipsilateral | 37.09 | 45.85 | 51.81 | 52.13 |
| Far Ipsilateral | 38.74 | 37.71 | 54.18 | 42.53 |

**Supplementary Table 1.** Shot 1 accuracy and Trial Success for the two elevations and five directions. Values were computed as the mean across 20 subjects, collapsed across all seven blocks.

| | Shot 1 Accuracy (%) | Trial Success (%) |
|---|---|---|
| **Block 1** | 33.31 | 41.85 |
| **Block 2** | 39.24 | 49.37 |
| **Block 3** | 38.19 | 50.31 |
| **Block 4** | 40.56 | 52.83 |
| **Block 5** | 41.49 | 53.81 |
| **Block 6** | 45.71 | 58.21 |
| **Block 7** | 47.47 | 60.05 |

**Supplementary Table 2.** Shot 1 accuracy and Trial Success for all Blocks. Values were computed as the mean across 20 subjects, collapsed across the ten target trajectories.
